# A phase IIb randomized, chronic-dosing, incomplete block, cross-over study of glycopyrronium, delivered via metered dose inhaler, compared with a placebo and an active control in patients with moderate-to-severe COPD

**DOI:** 10.1186/s12931-018-0739-6

**Published:** 2018-03-05

**Authors:** Edward M. Kerwin, Selwyn Spangenthal, Christine Kollar, Earl St Rose, Colin Reisner

**Affiliations:** 1 Clinical Research Institute of Southern Oregon, Medford, OR USA; 2 American Health Research, Charlotte, NC USA; 3 Everest Clinical Research, Little Falls, NJ USA; 4 Pearl – A member of the AstraZeneca Group, Morristown, NJ USA; 5 AstraZeneca, Gaithersburg, MD USA

**Keywords:** Bronchodilator, Chronic obstructive pulmonary disease, Co-suspension delivery technology, Glycopyrronium, Long-acting β_2_-agonist, Long-acting muscarinic antagonist, Metered dose inhaler

## Abstract

**Background:**

Long-acting muscarinic antagonist (LAMA) and long-acting β_2_-agonist (LABA) bronchodilators are key to the pharmacologic treatment of chronic obstructive pulmonary disease (COPD). This Phase IIb study investigated the safety and efficacy of four doses of the LAMA glycopyrronium (GP) delivered using co-suspension delivery technology via metered dose inhaler (MDI). The study was part of a wider clinical trial program performed to determine the optimal dose of GP MDI, the LABA formoterol fumarate dihydrate (FF) MDI, and glycopyrronium/formoterol fumarate dihydrate (GFF) MDI fixed-dose combination to be taken forward into Phase III studies.

**Methods:**

In this randomized, double-blind, 7-day chronic-dosing, three-period incomplete block, cross-over study, patients with moderate-to-severe COPD received two of the four doses of GP MDI (28.8 μg, 14.4 μg, 7.2 μg, and 3.6 μg) twice daily (BID), and either placebo MDI BID or open-label ipratropium MDI 34 μg four times daily. The primary efficacy endpoint was forced expiratory volume in 1 s (FEV_1_) area under the curve from 0 to 12 h (AUC_0–12_) relative to baseline on Day 7. Secondary and exploratory efficacy endpoints were assessed on Days 1 and 7. Safety and tolerability were evaluated throughout the study.

**Results:**

All GP MDI treatments were superior to placebo MDI for the primary efficacy endpoint (all *p* < 0.0001). However, only GP MDI 28.8 μg and 14.4 μg demonstrated statistical superiority to placebo MDI for all secondary efficacy endpoints analyzed in this study, with the exception of GP MDI 14.4 μg versus placebo MDI for the proportion of patients achieving ≥12% improvement in FEV_1_. No nominally significant differences were observed between GP MDI 28.8 μg and GP MDI 14.4 μg for any of the endpoints. All doses of GP MDI were well tolerated, with no unexpected safety findings.

**Conclusions:**

This study indicated that there was no advantage of GP MDI 28.8 μg compared with GP MDI 14.4 μg. It therefore added to the evidence from the Phase I/II clinical trial program, which identified GP MDI 14.4 μg as the most appropriate dose for use in the Phase III clinical studies.

**Trial registration:**

ClinicalTrials.gov (NCT01350128). Registered May 09, 2011.

## Background

Chronic obstructive pulmonary disease (COPD) is a commonly occurring disease characterized by persistent respiratory symptoms and airflow limitation [1]. The treatment of stable COPD aims to decrease both symptoms and the risk of exacerbations in patients, and mainly relies on the use of long-acting muscarinic antagonist (LAMA) and long-acting β_2_-agonist (LABA) bronchodilators [1].

Glycopyrronium/formoterol fumarate dihydrate (GFF) metered dose inhaler (MDI) 14.4/10 μg (Bevespi Aerosphere®; equivalent to glycopyrrolate/formoterol fumarate 18/9.6 μg) is a dual LAMA/LABA fixed-dose combination (FDC) therapy formulated using innovative co-suspension delivery technology, which enables the uniform delivery of multiple treatments in a single inhaler [2–4]. GFF MDI is now approved in the USA for the long-term maintenance treatment of airflow obstruction in patients with COPD [5].

This study was part of a wider Phase I/II clinical trial program investigating the safety and efficacy of glycopyrronium (GP) MDI, formoterol fumarate dihydrate (FF) MDI, and GFF MDI, all formulated using the same innovative co-suspension delivery technology [6–11]. The overall aim of this program was to determine the optimal dose of GP MDI, FF MDI, and GFF MDI to be taken forward into the Phase III clinical trials, PINNACLE-1 and PINNACLE-2 [12], and the 28-week safety extension study, PINNACLE-3 [13].

The objective of this Phase IIb, randomized, placebo-controlled, double-blind, incomplete block, cross-over study was to assess the safety and efficacy of GP MDI across the dose range 3.6 μg to 28.8 μg twice daily (BID) after 7-day dosing, compared with placebo MDI BID and open-label ipratropium bromide MDI (Atrovent® hydrofluoroalkane [HFA]) four times daily (QID) in patients with moderate-to-severe COPD. The doses selected for evaluation in this study were based on the previous findings with GP MDI for doses from 14.4 μg to 115.2 μg [8, 9].

## Methods

### Study population

The study population comprised male and female patients (40 to 80 years of age), who were current or former smokers with a history of at least 10 pack-years of cigarette smoking. Eligible patients were required to have a diagnosis of COPD as defined by the American Thoracic Society (ATS)/European Respiratory Society [14], with a severity defined as a pre- and post-bronchodilator forced expiratory volume in 1 s (FEV_1_)/forced vital capacity (FVC) < 0.70 at screening, and a post-bronchodilator FEV_1_ ≥ 30% and < 80% of predicted normal and ≥750 mL at screening, and a pre-bronchodilator FEV_1_ < 80% of predicted normal at baseline. Laboratory tests and electrocardiogram (ECG) performed at screening, and chest X-ray or computerized tomography (CT) scan performed within 6 months prior to screening, had to be deemed acceptable by the investigator for inclusion in the study.

Patients with not stable, exacerbating COPD, defined as acute worsening of COPD that required hospitalization in the 3 months prior to screening or the use of corticosteroids (parenteral or oral) or antibiotics in the 6 weeks prior to or during screening were excluded from participation in the study. Patients with a primary diagnosis of asthma, those who had alpha-1 antitrypsin deficiency, those who had undergone a lung resection, or those who had other respiratory disorders that may have impacted on the study were also excluded. Additionally, patients who had lower respiratory tract infections that required antibiotics within 6 weeks prior to screening, and patients who could not perform acceptable spirometry, were not eligible for inclusion in the study. Pregnant or lactating women, patients with a known or suspected history of substance abuse in the past 2 years, those with a history of hypersensitivity to short-acting or long-acting β_2_-agonists or muscarinic antagonists, or any component of the MDI, or patients who had clinically significant medical conditions (including, but not limited to, cardiovascular, neurological, psychiatric, hepatic [including liver function test abnormalities], gastrointestinal, immunological, glaucoma, symptomatic prostatic hypertrophy, endocrine [including uncontrolled diabetes or thyroid disease], hematological medical problems, urinary retention problems [including bladder-neck obstruction, i.e. difficulty passing urine, painful urination], uncontrolled hypertension, cancer not in complete remission for ≥5 years, creatinine clearance ≤50 mL/min, chest X-ray/CT scan abnormalities and ECG abnormalities) were also excluded. Patients taking prohibited medications, or those who were medically unable to withhold their short-acting bronchodilators for the 6-h period required prior to spirometry testing at each study visit, were also excluded from participation in this study. Furthermore, if patients were receiving long-term or nocturnal oxygen therapy for more than 12 h per day, if they had participated in the acute phase of a pulmonary rehabilitation program within 4 weeks prior to screening, or if they would enter the acute phase of a pulmonary rehabilitation program during the study, they were not eligible for inclusion. If patients required the use of a spacer to compensate for poor hand-to-breath coordination with an MDI, or if they had received treatment with an investigational study drug or had participated in another clinical study within the last 30 days or five half-lives prior to screening, they were also excluded.

Patients were willing and able, in the opinion of the investigator, to change current COPD medication, were able to comply with study procedures and to remain at the study center as required, and had agreed to take acceptable contraceptive precautions during the study, where appropriate.

### Study design

**Fig. 1:**
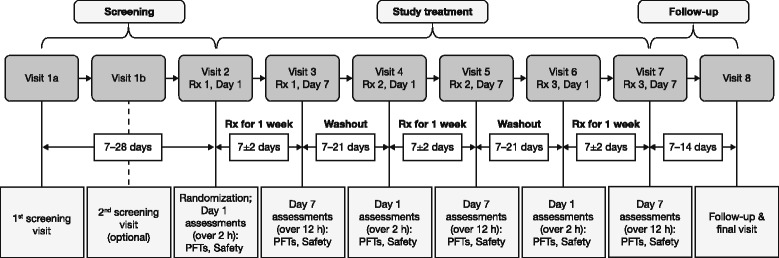
Study design. *PFT* Pulmonary function test, *Rx* Treatment

This was a 7-day, chronic-dosing, Phase IIb study with a randomized, double-blind, three-period, six-treatment, placebo-controlled, incomplete block, cross-over design, conducted in patients with moderate-to-severe COPD across nine sites in the USA between May 12, 2011 and October 04, 2011 (Fig. 1). Eligible participants were randomly assigned to one of 72 treatment sequences using a centralized interactive web response system. Each sequence included three of the six treatment groups (two doses of GP MDI and either placebo MDI or ipratropium MDI): GP MDI 28.8 μg, 14.4 μg, 7.2 μg, and 3.6 μg ex-actuator delivered as two actuations, BID; placebo MDI delivered as two actuations, BID; and open-label ipratropium bromide MDI 34 μg, as the active control, delivered as two actuations, QID.

Patients underwent a washout period of 7 to 28 days before randomization. Patients reported to the clinic on Day 1 of each treatment period, and were discharged after all scheduled assessments had been completed. Patients reported to the clinic again on the last day of each treatment period (Day 7), and were discharged after all scheduled assessments had been completed. The treatment period lasted for 7 ± 2 days. Each treatment period was separated by a washout period of 7 to 21 days. Patients underwent a total of three treatment periods (Fig. 1). On the final clinic visit (7 to 14 days after the end of the third treatment period), patients underwent post-study assessments, including a final physical examination and safety assessments (Fig. 1).

Patients were permitted to use albuterol sulfate 90 μg (Ventolin® HFA) for relief of COPD symptoms during each treatment period, if required. Patients were permitted to use albuterol MDI, ipratropium MDI, or albuterol/ipratropium combination MDI during each washout period, in accordance with recommendations from the investigator. Patients who received an inhaled corticosteroid (ICS) as part of an FDC therapy containing fluticasone and salmeterol, mometasone and formoterol, or formoterol and budesonide, and had been maintained on a stable dose for at least 4 weeks, were switched to the corresponding dose of fluticasone, mometasone, or budesonide, administered as a single agent with albuterol MDI, ipratropium MDI, or albuterol/ipratropium combination MDI at the investigator’s discretion. Patients who received ICS that was not administered as an FDC together with a LABA, and had been maintained on a stable dose for at least 4 weeks, were permitted to continue the ICS. Protocol-adjusted ICS therapy was continued and remained stable throughout the study. All COPD medications, including ICS, were withheld for at least 6 h prior to each clinic visit, or the visit was rescheduled as soon as was practical but within the specified visit windows.

Patients were not allowed to consume grapefruit or grapefruit juice throughout the study and were not allowed xanthine-containing foods or beverages such as coffee, tea, chocolate, and cola (decaffeinated beverages were allowed) for at least 6 h prior to – and for the duration of – each clinic visit. Patients were required to refrain from smoking for at least 4 h prior to – and throughout the duration of – each clinic visit. Patients were permitted to use various nicotine-replacement treatments (such as chewing gum and patches) as needed*,* in accordance with recommendations from the investigator, during the entire clinic visit.

This study was conducted in accordance with Good Clinical Practice Guidelines including the International Conference on Harmonisation, the US Code of Federal Regulations, and the Declaration of Helsinki. An institutional review board (Independent Investigational Review Board, Inc., FL, USA; IRB00003563) approved the protocol and informed consent form, and written informed consent was obtained from patients prior to screening. The study was registered on the US National Institutes of Health’s ClinicalTrials.gov website (NCT01350128).

### Efficacy endpoints

The primary efficacy endpoint was FEV_1_ area under the curve from 0 to 12 h (AUC_0–12_) relative to baseline on Day 7. The secondary efficacy endpoints evaluated on Day 1 relative to baseline were: peak change in FEV_1_ (defined as highest value of FEV_1_ post-dose minus baseline); time to onset of action (≥10% improvement in mean FEV_1_); proportion of patients achieving ≥12% improvement in FEV_1_; and peak improvement in inspiratory capacity (IC; mean of 1- and 2- h post-dose values minus baseline). The secondary efficacy endpoints evaluated on Day 7 were: change from baseline in morning pre-dose FEV_1_ (defined as the average of the 60- and 30-min pre-dose values on Day 7 minus baseline); peak change from baseline in FEV_1_ (defined as highest value of FEV_1_ post-dose minus baseline); peak change from baseline in IC (mean of 1- and 2-h post-dose assessments minus baseline); and change from baseline at post-dose trough FEV_1_ (post-dose trough FEV_1_ defined as the mean of the FEV_1_ assessments taken at 11.5 and 12 h post-dose minus baseline). Exploratory efficacy endpoints on Day 7 included change from baseline in mean morning and evening pre- and post-dose daily peak flow readings taken by patients and recorded in patient diaries.

### Efficacy assessments

Pulmonary function tests including FEV_1_ and FVC, peak expiratory flow rate (PEFR), and slow vital capacity for IC were carried out using a spirometer that met or exceeded the minimum performance recommendations of the ATS, and were performed in accordance with ATS criteria [15]. All sites were provided with identical spirometry systems (KoKo® Spirometer, nSpire Health, Inc., Louisville, Colorado, USA) and all the study staff responsible for performing pulmonary function testing received standardized training.

On Day 1 of each treatment period, spirometry was performed at 1 h and at 30 min pre-dose, followed by 15 and 30 min, and 1 and 2 h post-dose. On Day 7 of each treatment period, spirometry was performed at 1 h and 30 min pre-dose, followed by 15 and 30 min, and 1, 2, 4, 5.5, 6.5, 8, 10, 11.5, and 12 h post-dose. The average of the two pre-dose assessments was used to establish Day 7 pre-dose FEV_1_ and FVC. On Day 1 of each treatment period, IC assessments were obtained at 1 h and at 30 min pre-dose, and at 1 and 2 h post-dose. On Day 7 of each treatment period, IC assessments were obtained at 1 h and at 30 min pre-dose, followed by 1, 2, 11.5, and 12 h post-dose. IC assessments preceded spirometry assessments. All patients were instructed on the performance of the IC maneuver. For the efficacy endpoints, baseline was defined as the mean of pre-dose values across Day 1 of each treatment period, where the pre-dose values for each visit day were averaged, and then all visit means were averaged. The baseline FEV_1_ on Day 1 of Treatment Periods 2 and 3 had to be within ±15% or 150 mL of the baseline FEV_1_ obtained on Day 1 of Treatment Period 1, or either the visit was rescheduled at the investigator's discretion or the patient was discontinued from the study.

At screening, patients were instructed on the use of a peak-flow meter to measure pre- and post-dose morning and evening peak flow rate at home. Peak flow rate was measured immediately before and 30 min after taking the study medication. Patients were required to complete diaries recording the actual time of dosing and home peak flow rate measurements. Diaries were provided at screening and on Day 1 of each Treatment Period, were completed daily by the patient, and were returned at the next visit. For the change from baseline in mean morning pre- and post-dose daily peak flow rate on Day 7, readings taken pre-dose on Day 1 of Treatment Period 1 were excluded.

### Safety evaluations

The safety profile of the study treatments was determined from physical examination findings, vital signs (including heart rate and blood pressure), clinical laboratory values (including hematology and chemistry) and 12-lead ECGs. These assessments were conducted at screening and final follow-up visit, and for up to 2 h post-dose on Day 1 and up to 12 h post-dose on Day 7 of each treatment period (physical examinations were conducted at screening and final follow-up only). Adverse events (AEs) were recorded at screening, on Day 1 and Day 7 of each treatment period and at final follow-up visit. AEs of interest were paradoxical bronchospasm and dry mouth.

### Statistical analyses

The safety population included all patients who were randomized, received at least one dose of any study medication, and had at least one post-dose safety assessment for that treatment. The intent-to-treat (ITT) population included all patients who were randomized, received at least one dose of a study medication, and had both baseline and post-baseline efficacy data for that study treatment. The modified intent-to-treat (mITT) population included all patients who completed at least two treatment periods, with at least one pre-dose assessment on Day 7 for each of these two treatment periods, and no protocol deviations that could have impacted efficacy results. The per-protocol (PP) population included all patients in the ITT population who completed all three treatment periods with at least 11.5 h of evaluable spirometry data on Day 7, and excluded any measurements that were excluded from the mITT population.

The primary efficacy analysis based on the primary efficacy endpoint (FEV_1_ AUC_0–12_ relative to baseline on Day 7), involved four *a priori* treatment comparisons for superiority of each of the four GP MDI treatments compared with placebo MDI. For the primary efficacy objective, strong control of the family-wise Type I error was achieved by hierarchical testing according to dose order, from the highest dose to the lowest dose [16]. The least squares mean (LSM), difference in LSM, and associated standard errors (SEs) and two-sided 95% confidence intervals (CIs) were based on a linear mixed-effect model with FEV_1_ AUC_0–12_ (dependent-variable) and the following factors: baseline FEV_1_ (covariate), patient (sequence) (a random factor), period, sequence, treatment, and prior treatment (carry-over). The mITT population was the primary analysis population for the efficacy endpoints. Sensitivity analyses on the primary efficacy endpoint were carried out in the ITT and PP populations.

Secondary efficacy analysis of the primary efficacy endpoint involved comparisons of each treatment group to open-label ipratropium MDI, which assumed a non-inferiority margin of 0.1 L. These comparisons were performed using the same mixed model, algorithms, and hierarchical testing strategy as for the primary efficacy analysis. Non-inferiority for a comparison was supported only if the lower bound of the 95% CI for the difference of GP MDI minus ipratropium MDI was greater than − 0.1 L. Other secondary efficacy analyses involved primary efficacy comparisons (superiority of each treatment group to placebo MDI) and secondary efficacy comparisons (non-inferiority of each treatment group to ipratropium MDI) on secondary endpoints. The secondary and the exploratory efficacy objectives were analyzed using the same mixed model as used for the primary analysis, with baseline and prior treatment as a covariate where appropriate, with the exception of time to onset of action which was analyzed using Murray’s method for weighted Kaplan-Meier statistics for paired data [17], and proportion of patients achieving ≥12% improvement in FEV_1_ which was analyzed using McNemar’s test. Two-sided 95% CIs were tabulated for endpoints analyzed using the mixed model. Testing for first-order carry-over effects using a mixed model was performed for secondary and exploratory analyses.

Power calculations were based on the primary efficacy endpoint, FEV_1_ AUC_0–12_ on Day 7 of each dosing period following administration of the study drug. A sample size of 100 randomized patients (84 completers) was planned to provide 90% power (assuming a significance test at the 5% level, with no multiplicity adjustment). For each efficacy comparison, between and within patient variance components were assumed to have standard deviations (SDs) of 0.13 L; the SE of each comparison was calculated using a generalized least squares analysis which assumed spherical errors with no carry-over effects. The non-centrality parameter of the t-test was calculated assuming the SE from the generalized least squares analysis and a difference of 0.1 L (the minimally clinically significant difference, which is defined as the change in pre-dose FEV_1_ that can be perceived by patients) [18].

## Results

### Study population

**Fig. 2:**
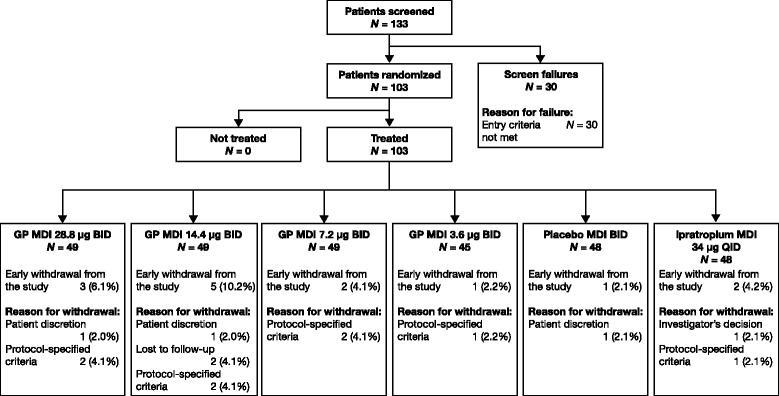
Patient disposition. *BID* Twice daily, *GP* Glycopyrronium, *MDI* Metered dose inhaler, *QID* Four times daily

A total of 133 patients were screened and 103 were randomized to receive study treatment (Fig. 2). Of these, 89 (86.4%) completed the study. All 103 randomized patients were included in the ITT and safety populations, 91 patients (88.3%) were included in the mITT population and 68 patients (66.0%) were included in the PP population. There were no clinically relevant differences in demographic or clinical characteristics among the patients according to treatment received. The mean age of the overall study population was 61.2 years, and 55 (53.4%) patients were male. The majority of study participants (88.3%) were of Caucasian race (Table 1). Overall, 69 (67.0%) patients were current smokers. Patients had smoked for an average of 64.2 pack-years, and had a mean duration of COPD at baseline of 8.1 years (Table 1). In the mITT population, 69.2% of patients had moderate COPD (post-bronchodilator FEV_1_ of ≥50% and < 80% and FEV_1_/FVC < 0.70) and 30.8% of patients had severe COPD (post-bronchodilator FEV_1_ of ≥30% and < 50% and FEV_1_/FVC < 0.70).

**Table 1:** Patient demographics and characteristics (ITT/safety population)

Parameter	GP MDI 28.8 μg BID, *N* = 49	GP MDI 14.4 μg BID, *N* = 49	GP MDI 7.2 μg BID, *N* = 49	GP MDI 3.6 μg BID, *N* = 45	Placebo MDI BID, *N* = 48	Ipratropium MDI 34 μg QID, *N* = 48	All patients, *N* = 103
Mean age, years (SD)	61.6 (7.9)	63.3 (7.9)	60.3 (8.2)	60.6 (8.8)	61.5 (7.8)	61.3 (9.0)	61.2 (8.2)
Gender, % male	49.0	55.1	59.2	48.9	60.4	43.8	53.4
Race, % Black or African/Caucasian/Other	6.1/85.7/8.2	8.2/87.8/4.1	8.2/87.8/4.1	6.7/88.9/4.4	8.3/87.5/4.2	6.3/87.5/6.3	6.8/88.3/4.9
Mean BMI, kg/m^2^ (SD)	30.1 (6.6)	27.5 (7.2)	29.0 (8.9)	29.0 (8.1)	28.2 (7.4)	29.2 (8.1)	28.8 (7.6)
Smoking status, % current smokers	63.3	59.2	67.3	71.1	64.6	66.7	67.0
Mean smoking history, pack-years (SD)	63.4 (25.8)	63.0 (28.2)	61.8 (27.5)	72.3 (28.0)	67.8 (30.1)	61.3 (25.9)	64.2 (27.4)
Mean duration of COPD at baseline, years (SD)	7.8 (4.8)	8.6 (6.8)	8.8 (5.5)	7.8 (5.8)	8.7 (6.5)	7.8 (5.5)	8.1 (5.9)
FEV_1_ ^a^, *N*	45	46	44	41	46	45	91
Mean screening FEV_1_ pre-bronchodilator, % predicted (SD)	49.9 (13.6)	47.0 (13.0)	48.3 (12.6)	47.4 (12.1)	47.8 (13.2)	48.6 (12.7)	48.2 (12.9)
Mean screening FEV_1_ pre-bronchodilator, L (SD)	1.464 (0.546)	1.379 (0.523)	1.471 (0.572)	1.407 (0.565)	1.474 (0.553)	1.406 (0.553)	1.440 (0.551)
Mean screening FEV_1_ post-bronchodilator, % predicted (SD)	57.8 (12.7)	55.0 (13.8)	56.2 (12.6)	56.1 (12.4)	55.3 (12.9)	57.3 (13.0)	56.3 (12.9)
Mean screening FEV_1_ post-bronchodilator, L (SD)	1.692 (0.536)	1.614 (0.584)	1.708 (0.598)	1.658 (0.613)	1.696 (0.566)	1.663 (0.611)	1.680 (0.586)
Mean baseline FEV_1_, % predicted (SD)	47.1 (14.1)	43.7 (13.3)	46.5 (12.3)	46.1 (12.4)	45.4 (14.0)	46.4 (12.3)	45.9 (13.2)
Mean baseline FEV_1_, L (SD)	1.381 (0.548)	1.285 (0.512)	1.430 (0.570)	1.372 (0.584)	1.404 (0.584)	1.346 (0.531)	1.376 (0.556)
Reversibility^a,b^, *N*	45	46	44	41	46	45	91
Mean reversibility post-bronchodilator for FEV_1_, % (SD)	18.3 (13.7)	18.5 (13.2)	18.1 (11.1)	19.9 (13.3)	17.4 (11.5)	19.8 (14.1)	18.6 (12.9)
Reversible, n (%)	28 (62.2)	30 (65.2)	28 (63.6)	28 (68.3)	31 (67.4)	28 (62.2)	59 (64.8)

^a^mITT population

^b^Reversibility was defined as > 200 mL improvement in FEV_1_ post-bronchodilator administration compared to pre-bronchodilator value and/or > 12% and > 150 mL improvement in FEV_1_ post-bronchodilator administration compared to pre-bronchodilator value

*BID* Twice daily, *BMI* Body mass index, *COPD* Chronic obstructive pulmonary disease, *FEV*_*1*_ Forced expiratory volume in 1 s, *GP* Glycopyrronium, *ITT* Intent-to-treat, *mITT* Modified intent-to-treat, *MDI* Metered dose inhaler, *QID* Four times daily, *SD* Standard deviation,

### Primary efficacy endpoint: FEV_1_ AUC_0–12_ on day 7

**Fig. 3:**
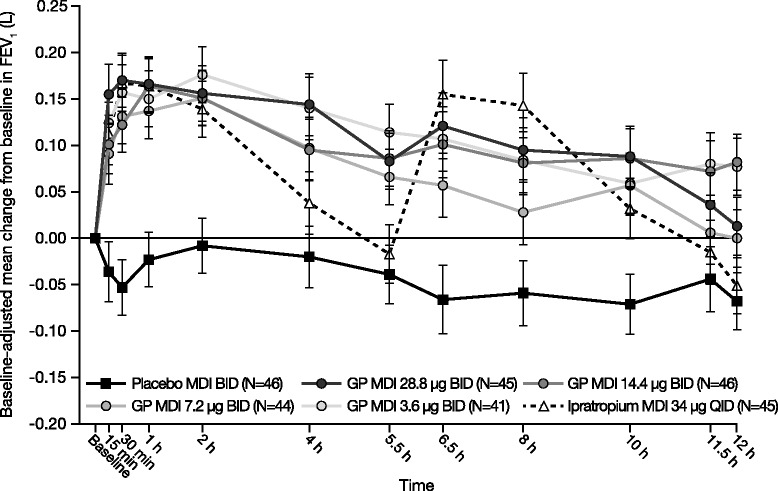
Mean change from baseline in FEV_1_ over time on Day 7 (mITT population). Error bars represent standard errors. *BID* Twice daily, *FEV*_*1*_ Forced expiratory volume in 1 s, *GP* Glycopyrronium, *MDI* Metered dose inhaler, *mITT* Modified intent-to-treat, *QID* Four times daily

**Fig. 4:**
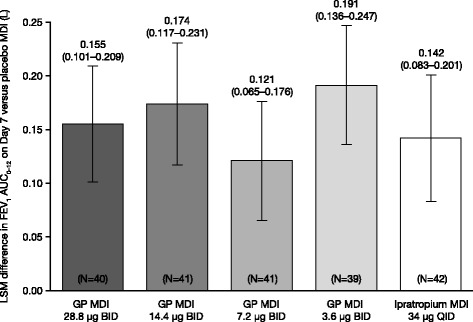
Adjusted difference from placebo in FEV_1_ AUC_0–12_ on Day 7 (mITT population). Error bars represent 95% confidence intervals. All *p* < 0.0001 versus placebo MDI. *AUC*_*0–12*_ Area under the curve from 0 to 12 h, *BID* Twice daily, *FEV*_*1*_ Forced expiratory volume in 1 s, *GP* Glycopyrronium, *LSM* Least squares mean, *MDI* Metered dose inhaler, *mITT* Modified intent-to-treat, *QID* Four times daily

GP MDI treatments showed a similar profile for FEV_1_ improvement over time, demonstrating an early onset of action to peak treatment effect within 2 h post-dose, followed by a gradual elimination over the 12-h period (Fig. 3). All GP MDI treatments were superior to placebo MDI, as measured by FEV_1_ AUC_0–12_ on Day 7 relative to baseline in the mITT population (Fig. 4). The estimated LSM differences versus placebo MDI for each GP MDI treatment ranged from 0.121 to 0.191 L (all *p* < 0.0001; Fig. 4). No clear dose ordering in the LSM differences from placebo MDI for FEV_1_ AUC_0–12_ on Day 7 was observed among the GP MDI treatments (Fig. 4). All GP MDI treatments were shown to be non-inferior to ipratropium MDI. When the GP MDI doses were compared, only GP MDI 7.2 μg demonstrated a smaller treatment effect than GP MDI 3.6 μg (LSM difference: − 0.071 L; *p* = 0.0127). The results of the sensitivity analyses of the primary efficacy endpoint performed in the ITT and PP populations were consistent with the findings in the mITT population.

### Secondary efficacy endpoints evaluated on day 1

All GP MDI doses demonstrated superiority to placebo MDI for peak change from baseline in FEV_1_ and IC, and a higher proportion of patients achieved ≥10% improvement from baseline in FEV_1_ within 30 min and 2 h for all GP MDI doses compared with placebo MDI (Table 2). The two highest doses of GP MDI, 28.8 μg and 14.4 μg, showed the largest benefits compared with placebo MDI for peak change in IC. All GP MDI doses had a faster onset of action than placebo (mean difference: − 0.37 to − 0.79 h, *p* ≤ 0.0045). A significantly higher proportion of patients achieved ≥12% improvement in FEV_1_ when receiving GP MDI 28.8 μg, 7.2 μg, and 3.6 μg compared with placebo MDI (Table 2). However, no clear dose ordering was observed among GP MDI doses for any of the secondary efficacy endpoints evaluated on Day 1 (Table 2). When the GP MDI doses were compared, peak change from baseline in FEV_1_ was greater with GP MDI 28.8 μg compared with GP MDI 7.2 μg (LSM difference: 0.064 L, *p* = 0.0335), and peak change from baseline in IC was greater with GP MDI 14.4 μg compared with GP MDI 7.2 μg (LSM difference: 0.091 L, *p* = 0.0427). Additionally, GP MDI 28.8 μg had a faster onset of action compared with GP MDI 7.2 μg and 3.6 μg (mean differences: − 0.42 and − 0.27 h, respectively, *p* ≤ 0.0362).

**Table 2:** Secondary efficacy endpoints on Day 1 (mITT population)

	GP MDI 28.8 μg BID	GP MDI 14.4 μg BID	GP MDI 7.2 μg BID	GP MDI 3.6 μg BID	Placebo MDI BID	Ipratropium MDI 34 μg QID
Peak change from baseline in FEV_1_ ^a^, L						
*N*	45	46	44	41	46	44
LSM	0.245^†^	0.221^†^	0.181^***^	0.204^†^	0.071	0.250^†^
95% CI	0.195–0.295	0.172–0.270	0.130–0.232	0.153–0.255	0.021–0.120	0.198–0.301
Time to onset of action (proportion of patients achieving ≥10% improvement from baseline in FEV_1_), %						
*N*	44	45	43	40	45	44
Within 30 min	63.6	48.9	25.6	45.0	15.6	72.7
Within 2 h	70.5	73.3	62.8	67.5	33.3	84.1
Patients achieving ≥12% improvement in FEV_1_, %						
*N*	45	46	44	41	46	45
Proportion of patients	66.7^**^	58.7	52.3^**^	61.0^***^	17.4^b^	77.8
Peak change from baseline in IC^c^, L						
*N*	45	45	44	41	46	45
LSM	0.240^†^	0.276^†^	0.185^**^	0.187^**^	0.040	0.252^†^
95% CI	0.165–0.316	0.201–0.351	0.111–0.259	0.111–0.262	−0.036–0.117	0.173–0.330

95% CIs presented are for each individual treatment (not versus placebo MDI)

^**^p ≤ 0.01, ^***^p ≤ 0.001, ^†^*p* ≤ 0.0001 compared with placebo MDI

^a^Highest value of FEV_1_ post-dose on Day 1 minus baseline, where baseline = average of FEV_1_ pre-dose values across Day 1 of each treatment period

^b^No pairs

^c^Mean of 1 and 2 h post-dose on Day 1 minus baseline, where baseline = average of IC pre-dose values across Day 1 of each treatment period

*BID* Twice daily, *CI* Confidence interval, *FEV*_*1*_ Forced expiratory volume in 1 s, *GP* Glycopyrronium, *IC* Inspiratory capacity, *LSM* Least squares mean, *MDI* Metered dose inhaler, *mITT* Modified intent-to-treat, *QID* Four times daily

### Secondary efficacy endpoints evaluated on day 7

All GP MDI doses demonstrated superiority to placebo MDI for peak change from baseline in FEV_1_ and IC (Table 3). Only GP MDI 28.8 μg and 14.4 μg demonstrated superiority to placebo MDI for change from baseline in morning pre-dose FEV_1_ and at 12-h post-dose trough FEV_1_ (Table 3). Based on the hierarchical testing strategy, no claim could be made for the superiority of GP MDI 3.6 μg to placebo MDI for these endpoints, because change from baseline in morning pre-dose FEV_1_ and at 12-h post-dose trough FEV_1_ for GP MDI 7.2 μg versus placebo MDI were not statistically significant. No clear dose ordering was observed among GP MDI doses for any of the secondary efficacy endpoints evaluated on Day 7 (Table 3). All GP MDI doses led to numerically greater changes from baseline in morning pre-dose FEV_1_ and 12-h post-dose trough FEV_1_ than ipratropium MDI. When GP MDI doses were compared, GP MDI 28.8 μg, 14.4 μg, and 3.6 μg had a greater mean change from baseline in morning pre-dose FEV_1_ than GP MDI 7.2 μg (LSM differences: 0.065 L to 0.078 L, *p* ≤ 0.0373).

**Table 3:** Secondary and exploratory efficacy endpoints on Day 7 (mITT population)

	GP MDI 28.8 μg BID	GP MDI 14.4 μg BID	GP MDI 7.2 μg BID	GP MDI 3.6 μg BID	Placebo MDI BID	Ipratropium MDI 34 μg QID
Change from baseline in morning pre-dose FEV_1_ ^a^, L						
*N*	45	45	43	41	46	43
LSM	0.088^†^	0.075^†^	0.010	0.084^†^	−0.043	− 0.088
95% CI	0.039–0.136	0.027–0.124	−0.039–0.059	0.035–0.133	−0.092–0.006	−0.138 to −0.037
Peak change from baseline in FEV_1_ ^b^, L						
*N*	45	45	43	41	44	43
LSM	0.242^†^	0.224^†^	0.223^†^	0.238^†^	0.070	0.225^†^
95% CI	0.181–0.302	0.164–0.284	0.162–0.285	0.176–0.300	0.009–0.131	0.164–0.287
Peak change from baseline in IC^c^, L						
*N*	45	45	43	41	44	43
LSM	0.213^†^	0.189^†^	0.161^†^	0.192^†^	−0.029	0.191^†^
95% CI	0.130–0.295	0.107–0.271	0.077–0.245	0.107–0.277	−0.112–0.055	0.106–0.275
Change from baseline at 12-h post-dose trough FEV_1_ ^d^, L						
*N*	40	41	41	39	41	42
LSM	0.018^*^	0.079^†^	0.004	0.077^†^	−0.054	− 0.041
95% CI	−0.045–0.080	0.018–0.139	−0.057–0.065	0.016–0.139	−0.117–0.009	− 0.103–0.021
Change from baseline in mean morning pre-dose daily PEFR, L/min						
*N*	36	34	35	29	35	14
LSM	10.826^*^	14.230^**^	13.574^*^	2.582	−7.877	7.625
95% CI	−2.749–24.402	0.334–28.125	−0.274–27.423	−12.126–17.289	−21.785–6.032	−12.890–28.140
Change from baseline in mean morning post-dose daily PEFR, L/min						
*N*	32	35	31	27	32	6
LSM	18.618^***^	14.979^**^	19.976^***^	17.530^**^	−13.040	15.190
95% CI	3.490–33.747	0.987–28.970	4.777–35.174	1.929–33.131	−27.150–1.069	−16.092–46.472
Change from baseline in mean evening pre-dose daily peak flow rate, L/min						
*N*	35	31	33	26	32	11
LSM	14.002^**^	18.275^**^	10.610^*^	23.336^***^	−3.928	14.808
95% CI	1.564–26.440	5.220–31.330	−2.155–23.374	9.637–37.034	−16.751–8.895	−4.635–34.251
Change from baseline in mean evening post-dose daily peak flow rate, L/min						
*N*	19	16	20	17	20	8
LSM	22.701^*^	17.790	24.585^*^	36.885^*^	− 12.269	46.772^*^
95% CI	− 0.329–45.732	− 8.811–44.391	−0.947–50.117	9.152–64.619	− 35.536–10.998	7.415–86.130

95% CIs presented are for each individual treatment (not versus placebo MDI)

^*^p < 0.05, ^**^p ≤ 0.01, ^***^p ≤ 0.001, ^†^*p* ≤ 0.0001 compared with placebo MDI. Due to hierarchical testing, no claims were advanced for a GP MDI treatment vs. placebo MDI, unless all higher dose-levels of the GP MDI were superior to placebo MDI

^a^Defined as the average of the 60- and 30-min pre-dose values on Day 7 minus baseline (average across Day 1 of each treatment period)

^b^Defined as highest value of FEV_1_ post-dose minus baseline on Day 7, where baseline = average of pre-dose values across Day 1 of each treatment period

^c^Mean of 1- and 2-h post-dose assessments on Day 7 minus baseline, where baseline = average of IC pre-dose values across Day 1 of each treatment period

^d^Mean of the FEV_1_ assessments taken at 11.5 and 12 h post-dose minus baseline, where baseline = average of FEV_1_ pre-dose values across Day 1 of each treatment period

*BID* Twice daily, *CI* Confidence interval, *FEV*_*1*_ Forced expiratory volume in 1 s, *GP* Glycopyrronium, *IC* Inspiratory capacity, *LSM* Least squares mean, *MDI* Metered dose inhaler, *mITT* Modified intent-to-treat, *QID* Four times daily

### Exploratory efficacy endpoints on day 7

GP MDI 28.8 μg, 14.4 μg, and 7.2 μg demonstrated superiority to placebo MDI for change from baseline in mean morning pre-dose daily peak flow rate, whereas all GP MDI doses were superior to placebo MDI post-dose (Table 3). For change from baseline in mean evening daily peak flow rate, all GP MDI doses were superior to placebo MDI pre-dose, whereas only GP MDI 28.8 μg demonstrated superiority to placebo MDI post-dose (Table 3). Based on the hierarchical testing strategy, no claim could be made for superiority of GP MDI 7.2 μg and 3.6 μg to placebo MDI for this endpoint, because change from baseline in mean evening post-dose daily peak flow rate for GP MDI 14.4 μg versus placebo MDI was not statistically significant. There were no differences when comparisons were made between GP MDI doses.

### Safety

Overall, 45 patients (43.7%) reported at least one treatment-emergent AE (TEAE) at any time during the study (GP MDI: 14.3–28.6%; placebo MDI, 14.6%; ipratropium MDI, 31.3%; Table 4). A total of 21 patients (20.4%) reported TEAEs related to the study treatment (GP MDI: 6.1–14.3%; placebo MDI, 6.3%; ipratropium MDI, 14.6%; Table 4). No TEAEs led to early withdrawal from the study and no deaths were reported.

**Table 4:** Summary of TEAEs (safety population)

Parameter	GP MDI 28.8 μg BID, *N* = 49	GP MDI 14.4 μg BID, *N* = 49	GP MDI 7.2 μg BID, *N* = 49	GP MDI 3.6 μg BID, *N* = 45	Placebo MDI BID, *N* = 48	Ipratropium MDI 34 μg QID, *N* = 48	All patients, *N* = 103
Patients with at least one TEAE, *n* (%)	7 (14.3)	11 (22.4)	14 (28.6)	10 (22.2)	7 (14.6)	15 (31.3)	45 (43.7)
Patients with TEAEs related to study treatment, *n* (%)	3 (6.1)	4 (8.2)	7 (14.3)	6 (13.3)	3 (6.3)	7 (14.6)	21 (20.4)
Patients with SAEs, *n* (%)	0	0	0	1 (2.2)	0	1 (2.1)	2 (1.9)
TEAEs reported in ≥2% of patients for any treatment arm, *n* (%) (preferred term)							
Dry mouth	1 (2.0)	2 (4.1)	4 (8.2)	4 (8.9)	1 (2.1)	3 (6.3)	12 (11.7)
Cough	1 (2.0)	2 (4.1)	2 (4.1)	0	0	3 (6.3)	6 (5.8)
Headache	1 (2.0)	1 (2.0)	1 (2.0)	2 (4.4)	0	0	4 (3.9)
Diarrhea	1 (2.0)	0	1 (2.0)	0	0	1 (2.1)	3 (2.9)
Dyspnea	0	1 (2.0)	0	0	2 (4.2)	0	3 (2.9)
Hypertension	0	0	2 (4.1)	1 (2.2)	0	0	3 (2.9)
Nasopharyngitis	0	1 (2.0)	1 (2.0)	0	0	1 (2.1)	3 (2.9)
Oropharyngeal pain	0	1 (2.0)	0	0	0	2 (4.2)	3 (2.9)
Pyrexia	0	0	0	0	1 (2.1)	2 (4.2)	3 (2.9)
Vomiting	1 (2.0)	0	1 (2.0)	1 (2.2)	1 (2.1)	1 (2.1)	3 (2.9)

*BID* Twice daily, *GP* Glycopyrronium, *MDI* Metered dose inhaler, *QID* Four times daily, *SAE* Serious adverse event, *TEAE* Treatment-emergent adverse event

The most commonly reported TEAEs were dry mouth (11.7%), cough (5.8%), and headache (3.9%). Other TEAEs reported in at least 2% of patients overall were diarrhea, vomiting, dyspnea, oropharyngeal pain, nasopharyngitis, pyrexia, and hypertension (2.9% each; Table 4). There were no clinically relevant differences in the occurrence of TEAEs across treatments. Furthermore, there was no dose relationship with these TEAEs, with dry mouth occurring most frequently in patients treated with GP MDI 7.2 μg and 3.6 μg (four patients each; 8.2% and 8.9%, respectively), followed by in patients in the ipratropium MDI treatment group (three patients; 6.3%). No cases of paradoxical bronchospasm were observed.

Two patients (1.9%) reported serious AEs (Table 4) of COPD exacerbation (GP MDI 3.6 μg) and left-lower-extremity deep vein thrombosis (ipratropium MDI), both of which were considered unrelated to study treatment. No important trends were observed among the treatments in changes from baseline in clinical laboratory results, vital signs, and ECGs.

## Discussion

This Phase IIb, randomized, cross-over study assessed the efficacy and safety of GP MDI 28.8 μg, 14.4 μg, 7.2 μg, and 3.6 μg BID, in patients with moderate-to-severe COPD, compared with placebo MDI BID and open-label ipratropium MDI 34 μg QID.

For the primary efficacy endpoint, all doses of GP MDI demonstrated statistically significant and clinically relevant increases in FEV_1_ AUC_0–12_ compared with placebo MDI following 7 days of treatment, which is in agreement with findings for GP MDI 14.4 μg, 7.2 μg, and 3.6 μg following 14 days of treatment [6]. The short-acting muscarinic antagonist ipratropium is used as a standard comparator in early phase clinical studies with novel bronchodilators [19], and non-inferiority testing is commonly applied to compare the effects of bronchodilators with the active comparator. Non-inferiority to ipratropium MDI was observed for all doses of GP MDI for FEV_1_ AUC_0–12_ on Day 7. Additionally, for FEV_1_ improvement over time, the similarity of results between the first 5.5 h and the second 6.5 h with all GP MDI doses relative to ipratropium MDI provided further evidence supporting the appropriateness of BID dosing for GP MDI.

GP MDI 28.8 μg and 14.4 μg demonstrated superiority to placebo MDI for all secondary efficacy endpoints statistically analyzed in this study, with the exception of the proportion of patients achieving ≥12% improvement in FEV_1_ on Day 1 for which GP MDI 14.4 μg did not show superiority to placebo MDI. These results are comparable with a 14-day study, where GP MDI 14.4 μg showed superiority to placebo MDI for all secondary efficacy endpoints, with the exception of change in morning pre-dose trough FEV_1_ on Day 7, although GP MDI 14.4 μg did show superiority to placebo MDI for the proportion of patients achieving ≥12% improvement in FEV_1_ on Day 1 in this study [6].

No clear dose ordering was observed among the GP MDI doses. All GP MDI doses showed superiority to placebo MDI for three of the secondary endpoints on Day 1, time to onset of action and peak change in FEV_1_ and in IC. In addition, for two of the secondary endpoints on Day 7, change from baseline in morning pre-dose FEV_1_ and at 12-h post-dose trough FEV_1_, superiority to placebo MDI was demonstrated for GP MDI 28.8 μg and 14.4 μg but, due to the results with 7.2 μg and the hierarchical testing strategy, superiority could not be declared for GP MDI 3.6 μg. The observations are largely consistent with previous findings, where a dose response was observed across GP MDI doses from 0.5 μg to 3.6 μg for the primary efficacy endpoint of FEV_1_ AUC_0–12_ relative to baseline at Day 14, with a relatively flat dose-response curve for the higher doses of 7.2 μg and 14.4 μg. Moreover, no clear dose-response was observed for many of the secondary efficacy endpoints for doses lower than 14.4 μg [6].

The exploratory peak flow rate endpoints also showed improvements following treatment with GP MDI compared with placebo MDI, without clear dose ordering for the GP MDI dose.

All doses of GP MDI were well tolerated, with no unexpected safety findings. There were no withdrawals from the study due to TEAEs. The most frequent TEAE was dry mouth, which is a well-established AE associated with LAMAs due to their anticholinergic activity [20, 21].

Limitations of this study included the short 7-day treatment period, and the open-label nature of the ipratropium active control. Patient-reported outcomes, such as St George’s Respiratory Questionnaire and COPD assessment test scores, were not assessed due to the short timeframe. Additionally, patients eligible for inclusion in this study were not required to show reversibility to short-acting bronchodilators. An inclusion criteria of reversibility may have led to better dose-response ordering for GP MDI. However, as reversibility to short-acting bronchodilators does not influence treatment decisions in routine clinical practice, the absence of a requirement for reversibility may enhance the applicability of the findings of this study to real-world practice. A strength of this study was the cross-over design, although direct comparisons between ipratropium MDI and placebo MDI were not possible, as each patient received only one of these treatments.

The findings in this study did not demonstrate any advantage of GP MDI 28.8 μg compared with 14.4 μg in terms of lung function endpoints. Additionally, superiority to placebo MDI was demonstrated for GP MDI 3.6 μg for the primary efficacy endpoint and for some secondary efficacy endpoints, which indicated that this may not be the minimum effective dose. These results, therefore, supported further evaluation of GP MDI at doses below 3.6 μg and no higher than 14.4 μg BID, which were explored in a study that investigated the dose-response of GP MDI 14.4 μg, 7.2 μg, 3.6 μg, 1.9 μg, 1.0 μg, and 0.5 μg BID over 14 days and found that GP MDI 14.4 μg demonstrated the greatest efficacy versus placebo MDI, with no increase in the incidence of AEs [6]. The findings from these Phase IIb studies added to the evidence that resulted in GP MDI 14.4 μg BID being selected as the optimal dose for investigation in the Phase III studies PINNACLE-1, PINNACLE-2, and PINNACLE-3 [12, 13].

## Conclusions

The findings of this study demonstrated the efficacy and safety of 7-day dosing with GP MDI 3.6 μg to 28.8 μg BID for patients with moderate-to severe COPD. All doses of GP MDI in this study were well tolerated, and the most frequent TEAE observed (dry mouth) was a well-established AE associated with LAMA therapy. No unexpected safety findings were observed. The results further supported GP MDI 14.4 μg for use in the Phase III clinical studies.

## Abbreviations

AE: Adverse event
ATS: American Thoracic Society
AUC_0–12_: Area under the curve from 0 to 12 h
BID: Twice daily
CI: Confidence interval
COPD: Chronic obstructive pulmonary disease
CT: Computerized tomography
ECG: Electrocardiogram
FDC: Fixed-dose combination
FEV_1_: Forced expiratory volume in 1 s
FF: Formoterol fumarate dihydrate
FVC: Forced vital capacity
GFF: Glycopyrronium/formoterol fumarate dihydrate
GP: Glycopyrronium
HFA: Hydrofluoroalkane
IC: Inspiratory capacity
ICS: Inhaled corticosteroid
ITT: Intent-to-treat
LABA: Long-acting β_2_-agonist
LAMA: Long-acting muscarinic antagonist
LSM: Least squares mean
MDI: metered dose inhaler
mITT: Modified intent-to-treat
PEFR: Peak expiratory flow rate
PP: Per-protocol
QID: Four times daily
SAE: Serious adverse event
SD: Standard deviation
SE: Standard error
TEAE: Treatment-emergent adverse event
